# Factor-XIII activity in patients with mild to moderate ulcerative colitis and active bleeding: a prospective observational study

**DOI:** 10.1186/s13104-018-3963-8

**Published:** 2018-12-04

**Authors:** Karsten Bernerth, Ingolf Schiefke, Karin Liebscher, Susanne Raczynski, Tanja Kottmann, Niels Teich

**Affiliations:** 1 Practice for Internal Medicine, Leipzig, Germany; 2 Department of Gastroenterology Hepatology, Endocrinology, and Diabetology, Leipzig, Klinikum St. Georg gGmbH, Leipzig, Germany; 3 Institute of Transfusion Medicine and Clinical Hemostasis, Klinikum St. Georg gGmbH, Leipzig, Germany; 4 Practice for Gastroenterology, Markkleeberg, Germany; 5 Clinical Research Organization Dr. med Kottmann, Hamm, Germany; 6 Practice for Digestive and Metabolic Diseases, Nordstr. 21, 04105 Leipzig, Germany

**Keywords:** Factor-XIII activity, Ulcerative colitis, Inflammatory bowel disease

## Abstract

**Objective:**

Coagulation factor XIII plays a key role in fibrin clot stabilization and epithelial healing. Under chronic inflammatory conditions involving bleeding and an activation of the coagulation cascade, the FXIIIa inversely correlate with disease activity. We assumed that FXIIIa could be a predictor of severity in patients with ulcerative colitis (UC). Here, we evaluated the course of plasma activity of FXIIIa in 49 patients with mild to moderate UC and active rectal bleeding. Patients with a partial Mayo bleeding subscore > 2 were eligible to participate in our prospective observational study in an outpatient setting. FXIIIa was investigated during acute flare conditions, after bleeding had stopped and later on in quiescent UC.

**Results:**

Plasma activity of FXIIIa did not show any significant differences during the UC course. FXIIIa was measured below normal range < 70% in only 8 patients during the flare and increased to normal values during follow-up in 7 of these patients. Low FXIIIa during the flare was not associated with an increased bleeding activity. In patients with a mild to moderate UC flare and prolonged bleeding, FXIIIa activity is neither predictive of UC severity nor of any bleeding activity in an outpatient setting.

*Trial registration* This non interventional, non pharmacological prospective study was not obligated to receive a unique identifying number. This trial is registered with the Ethics Committee of the State Medical Chamber of Saxony, Dresden, Germany (Clinical Trials Registry number EK-BR-03/14-1)

## Introduction

Ulcerative colitis (UC) and Crohn’s disease (CD) are the two most common entities of inflammatory bowel diseases (IBD). The activity of clotting factor FXIII (FXIIIa) in IBD patients is inversely correlated with disease activity, as shown for both UC [1] and CD [2–6] and in further studies of both diseases ([7] and references within). One study from Italy evaluated the FXIII levels and their relationship with the clinical activity index, endoscopic score and histopathology among UC patients, and found negative correlations for all three assessments [8]. This is most probably attributable to an increased consumption of the factor in the ulcerated inflamed tissue and subsequent tissue regeneration [8].

The clinical effects of FXIII infusions on UC severity are uncertain as some studies provided evidence of a bleeding-stabilizing effect [9, 10] of FXIII administration whereas other authors found insignificant outcomes [11]. So far, the potential therapeutic effect of FXIII on mucosal healing in UC is still unknown. Nothing is known about the time-response of FXIII activity during a UC flare and whether low FXIII activity in the acute bleeding phase might be a predictor of severity. The aim of this study is to describe the course of FXIII activity during an acute UC flare in relation to bleeding activity.

## Main text

### Methods and analysis

Patients with mild to moderate active UC were eligible to participate in this study. UC activity was defined as a partial Mayo Score of ≥ 2–6 with a rectal bleeding subscore of ≥ 2. The partial Mayo Score is validated in order to define the clinical severity and the clinical response in UC [12] using the non-invasive components of the Mayo Score (stool frequency, bleeding components and physician assessment). Each of the three subscores range from 0 to 3, where higher scores indicate more severe disease.

In the past, all patients had to undergo a colonoscopy for histological confirmation of the diagnosis. Patients with concomitant bleeding disorder and anti-coagulating medications, patients under age 18 and pregnant women were excluded from the study.

In this prospective non-randomized observational study in an outpatient setting, we investigated the relevance of FXIIIa changes before, during and after an inflammatory UC flare. Patients were enrolled and assessed for FXIIIa on day 1 (visit 1, bleeding subscore ≥ 2), during recovery (visit 2, bleeding subscore < 2) and later on in quiescent disease (visit 3, bleeding subscore = 0). From April 2015 through May 2017, we conducted this trial in 3 IBD centers.

Outcomes included parameters such as clinical response, change in the partial Mayo score and in the bleeding subscore. We also examined changes from baseline in the absolute lymphocyte count and in the concentrations of C-reactive protein, calprotectin and coagulation values. In addition to clinical characteristics and laboratory data, the Short Form 36 (SF-36) questionnaire for health-related quality of life was obtained at all visits. Blood and stool samples were obtained at each visit. Hemostatic parameters such as prothrombin time (70–130%, Thromborel, Siemens Healthcare Diagnostics Products GmbH), activated partial thromboplastin time, aPTT (26.0–39.0 s, Pathrombin SL, Siemens Healthcare Diagnostics Products GmbH), thrombin time (14.0–19.0 s, STA-Thrombin, Diagnostica STAGO, S.A.S), fibrinogen (1.5–4.5 g/L, Multifibren*U, Siemens Healthcare Diagnostics Products GmbH), D–dimer (< 184 µg/L, TriniLIA Auto Dimer, Trinity Biotech GmbH), antithrombin (80–115%, Berichrom ATIII, Siemens Healthcare Diagnostics Products GmbH) and FXIIIa (70–140%, Berichrom FXIII by Siemens Healthcare Diagnostics Products GmbH) were recorded by use of automated coagulation analyzer BCS XP (Siemens Healthcare Diagnostics Products GmbH). The samples were sent to study laboratories separately, in temperature- and sunlight-protected receptacles, within 3 h and immediately centrifugated at 3000 rpm for 15 min and separated to 2–3 aliquots of 0.5 mL minimum and finally frozen to − 80 °C until analysis. The samples were then defrosted in a 37 °C warm water-bath for 4 min before undergoing automated measurement.

All analyses were done by approved laboratories according to the standard requirements and conditions for laboratory analysis of DIN EN ISO 15189.

On the basis of the known variance of FXIIIa from previous studies and assuming a conservative 25% intrasubject change as a clinically relevant change for FXIIIa for the mean ratio (visit 1 to visit 3), 67 subjects were considered sufficient for the 95% confidence interval (CI) to be within alpha error 5% with a power of 80%. An interim evaluation after inclusion of 50% of patients should be performed. K-Smirnow-test and Shapiro–Wilk-test were performed in order to analyze the normal distribution in variables. Repeated-measures analysis of variance (ANOVA) was performed in normal distributed variables; otherwise Friedman-test was applied for dependent samples with a 95% confidence interval (95% CI) by means of comparing scores and laboratory parameters recorded at the three visits. Spearman-correlation and significance of cohort were calculated in the 3 groups in order to identify significant relations between parameters. The SF-36 was calculated by summarizing the values obtained for the mentioned 8 categories, and T-score was performed to obtain results. The higher the obtained values, the lower the impairment status. Statistical analysis was executed in IBM-SPSS V.24.

Data are summarized by median and interquartile range for continuous variables or mean and standard deviation if appropriate (Mayo score and SF-36) and frequency counts for categorical variables. *P* values less than 0.05 were considered significant.

### Results

Out of 644 continuously treated UC patients at the enrolling sites, 52 patients were eligible for participation in our study from November 2014 through May 2017. 27 participants were female, 25 were male. The mean age was 38.4 (21–52) years for male and 35.6 (21–63) years for female patients, see Table 1. 49 patients completed the study and three were excluded for the following reasons: 1 patient withdrew participation and 2 patients had laboratory sample errors. Partial Mayo score fall from 5.2 (± 0.7) at baseline to 3.7 (± 2.2) at visit 2–2.3 (± 2.0) at visit 3 (p < 0.05).

**Table 1:** Demographic and baseline disease characteristics

Characteristics	
Age (year) (median (IQR))	35 (31–45)
Male sex, no (%)	25 (48)
Site of disease, no (%)	
Rectum and sigmoid colon	12 (23)
Left side colon	28 (54)
All of the colon	12 (23)

**Fig. 1:**
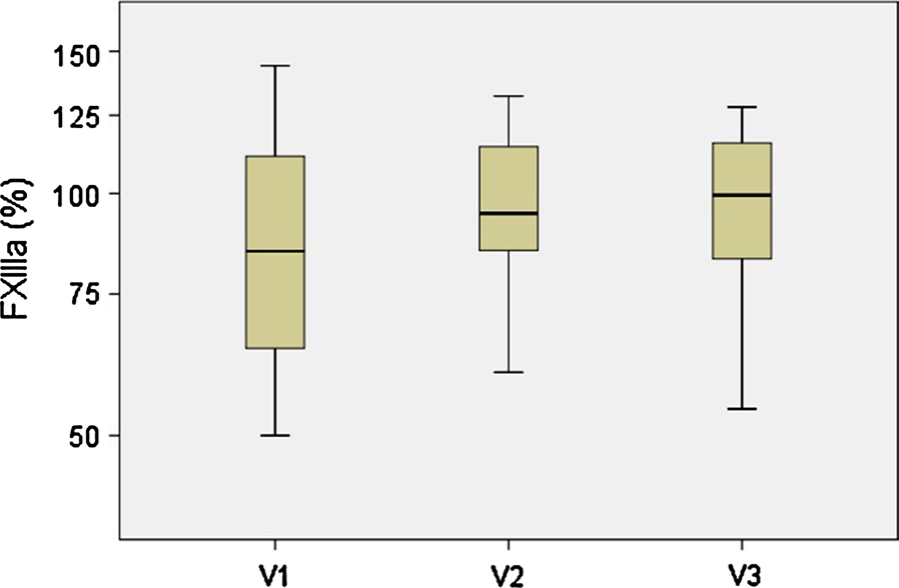
No correlation was found between FXIIIa and course of the UC flare (box-whisker-plot: median and interquartile range)

The measured FXIII activity was within the normal range in 41 participants (84%) at study inclusion and showed neither relevant changes during follow-up visits (Fig. 1) nor an association with the extent of UC involvement (proctitis, left sided UC and extended UC/pancolitis, data not shown). FXIIIa was measured below normal range < 70% in only 8 patients during the flare and increased to normal values during follow-up in these patients, except for 1 (not shown).

Low FXIIIa during the flare was not associated with the Mayo bleeding subscore (Spearman correlation coefficient 0.303, p = 0.366, not significant). Not even during longer UC episodes with elevated Mayo Bleeding Score (MBS) did FXIIIa show any significant changes. Only 1 patient showed a prolonged course of bleeding with continuously reduced FXIIIa and a significant positive correlation to CRP (Spearman correlation coefficient 0.317, p = 0.025).

The localization of the UC in our cohort showed a predominance in the left colon (N = 27 (55%), while pancolitis and proctitis were more rare in N = 11 subjects (22.4%). Analyzing correlation between UC localization and MAYO disease activity score, we found higher inflammatory activity in patients with proctitis than in patients with pancolitis or left side colitis [Pearsons correlation coefficient 0.286 (p = 0.047)].

Extreme-value analyses of the highest and lowest 5% in laboratory values showed a suspected inverse correlation of fibrinogen with FXIIIa (N = 5, p < 0.05) at visit 1 and visit 2 but lacked in sample size (Table 2). Inflammatory and haemostatic laboratory tests [i.e. antithrombin (p = 0.21), thrombin time (p = 0.13) and D-Dimer (p = 0.29)] did not meet any statistical significance as all as they were still too widely scattered. No correlation was found between fecal calprotectin and FXIII activity [Pearsons correlation coefficient − 0.11 (p = 0.96), variance analysis ANOVA (p = 0.28)].

**Table 2:** Laboratory results at baseline and during follow up visits

	Visit 1	Visit 2	Visit 3
	Median (IQR)	Median (IQR)	Median (IQR)
FXIIIa (%)	96.5 (77.7–115.0)	100.0 (85.0–113.5)	101 (86.5–121.5)
WBC (Gpt/L)	7.6 (5.9–9.5)	8.1 (6.5–11.1)	7.3 (5.9–9.2)
Hb (mmol/L)	8.4 (7.8–9.1)	8.4 (7.5–8.6)	8.1 (7.7–8.8)
PLT (Gpt/L)	307 (248–395)	312 (288–382)	302 (260–366)
Ferritin (ng/mL)	46.3 (21.9–146.0)	32.6 (11.9–55.6)	34 (14.7–93.8)
Transf. sat. (%)	17.7 (9.3–21.0)	17.9 (9.6–25.8)	19.4 (13.1–29.6)
CRP (mg/L)	13.1 (5.6–15.7)	4.4 (1.7–14.8)	1.3 (0.8–7.5)
Calprotectin (µg/g)	374.0 (98.0–1967.0)	272 (81.9–1336.2)	211 (65.5–522.5)
Thrombin Time (s)	14.6 (13.9–15.5)	14.8 (13.9–15.6)	14.9 (14.5–16.0)
Antithrombin (%)	104.0 (94.0–110.0)	98.0 (92.5–113.0)	101.0 (91.5–107.0)
Fibrinogen (g/L)	3.7 (2.9–4.4)	3.5 (2.4–3.9)	3.5 (2.4–4.0)
D–Dimer (µg/L)	199.0 (94.2–377.0)	164.0 (92.0–408.5)	129.0 (86.0–252.0)

The SF-36 analysis demonstrated a disability in physical health component summary score (PCS) at study enrollment with arithmetic mean values of 39.7 (SD 8.9; range 23.6–59.1) vs. 38.4 at visit 2 (10.0; 24.7–54.3) and 47.4 at visit 3 (9.3; 22.5–62.2), neither showing any significant differences in the Friedman-test (p = 0.3). However, these were lower than the mean value of a reference US population which was 48.4.

The mental health component summary score (MCS) was calculated at 42.5 for visit 1 (SD 10.4; 18.8–55.8), at 45.2 (SD 9.4; 25.8–60.2) for visit 2 and at 46.0 (SD 9.4; 28.3–58.2) for visit 3, p = 0.492. The US reference for MCS was 50.9 [13]. PCS and MCS showed a disability during the study period.

### Discussion

With our study, we firstly reported on FXIIIa activity during an acute flare of ulcerative colitis in a prospective well-defined outpatient cohort. The assumption that outpatients with a UC flare whose disease activity is characterized by hematochezia would show a decreased FXIIIa activity was disproved. Even in case of low FXIIIa at the beginning of the flare, we found no association with the bleeding severity during subsequent visits. Despite the long period between the visits and the small number of follow-up study participants this negative result of our study is well comparable to an earlier study by Linskens et al. [1] which was not able to demonstrate any significant relationship between a clinical course and coagulation parameters in hospitalized patients.

Our study-design, as initially suggested in an open label prospective FXIIIa study, did not show any significant differences during UC healing [11]. Another approach for our study was taken from Seitz et al., comparing FXIIIa and inflammation and coagulation in both, hospitalized UC and CD patients, assigned to active or moderate disease cohort [14]. Distal bowel involvement resulted in significantly higher FXIIIa than proximal affection, which contradicts our observation where proctitis resulted in lower FXIIIa. Follow-up results were obtained from 11 patients with active UC, and one case with FXIII supplementation was recorded: FXIII was higher at baseline in therapy-responders (mean 106%, N = 6] than in non-responders after day 14 (67.5%, N = 4). The correlation between a high endoscopic score and low FXIIIa was basically proven in this singular study, but the individual course was not representatively displayed and remains yet to be proven [14].

The examination of the inflammation, bleeding and coagulation parameters in our cohort shows that the respective parameters are essentially only slightly different from the normal values. Thus, it is clear that hematochezia, although characteristic of UC and threatening, leads to an overestimation of inflammatory activity. A recent publication reiterated the issue of UC activity assessment: although endoscopy with histology reveals a healed mucosa, diarrhea (up to 34%) and hematochezia (up to 24%) persist. The inflammation and bleeding parameters showed no deviation from the normal range [15]. This well compares to our results.

We conclude that FXIIIa activity is not predictive of UC severity in outpatients with mild to moderate UC flares, nor of any prolonged bleeding periods. Therefore a therapeutic approach for recombinant FXIIIa does not appear suitable for UC outpatients.

## Limitations

Bleeding and coagulation parameters including FXIIIa showed large standard deviations. The small number of follow-up study participants is another limiting factor for the data evaluation. Another limitation is the lack of patient assignment at the defined visit times and the long periods between visits, which make comparability between the cohorts of a visit more difficult.

## Abbreviations

CD: Crohns disease
IBD: inflammatory bowel diseases
UC: ulcerative colitis
MBS: Mayo Bleeding Score
MCS: mental health component summary score
PCS: physical health component summary score
